# Modifying water use practices to eliminate *Pseudomonas aeruginosa* bloodstream infections in the neonatal intensive care unit

**DOI:** 10.1017/ash.2023.223

**Published:** 2023-09-29

**Authors:** Ingrid Camelo, Srilatha Neshangi, Amy Thompson

## Abstract

**Objective:** To describe the strategies implemented at a tertiary-care healthcare center neopnatal intensive care unit (NICU) to control and assure prevention of subsequent central-line bloodstream infections (CLABSIs) with *Pseudomonas aeruginosa* after 4 cases of CLABSI with this organism were detected. **Methods:** During the months of September 2020 to February 2021, 4 cases of CLABSI with *Pseudomonas aeruginosa* were reported in our NICU in patients meeting criteria for extremely low birthweight (ELBW) infants. All patients were treated according to IDSA guidelines for management of bloodstream infections. To avoid the appearance of new events and to improve existing policies, we implemented a stepwise approach by reviewing routine disinfection and/or cleaning procedures of isolettes: (1) liners for bath basins were applied, (2) sterile water was provided for bathing newborns, (3) we ensured timely biomed preventive maintenance of water reservoirs for patient care equipment (nebulizers, isolettes and fluid warmers), and (4) we implemented the installment of point-of-care filters for tap water. **Results:** Measures were implemented from February 2021 to July 2021. During the following year from July 2021 to June 2022, no CLBSIs related to *Pseudomonas aeruginosa* were reported in our NICU in patients meeting criteria for ELBW infants. **Conclusions:** Recognition of CLABSI from organisms from water resources is important to implementing focused prevention strategies targeting water resources and water utilization practices. In our institution, these interventions yielded complete resolution, with no new infection events.

**Disclosure:** None

